# Virtual FFR Quantified with a Generalized Flow Model Using Windkessel Boundary Conditions

**DOI:** 10.1155/2020/3942152

**Published:** 2020-02-21

**Authors:** Keltoum Chahour, Rajae Aboulaich, Abderrahmane Habbal, Nejib Zemzemi, Chérif Abdelkhirane

**Affiliations:** ^1^LERMA, Mohammadia Engineering School, Mohamed V University in Rabat, Rabat, Morocco; ^2^Université Côte d'Azur, Inria, CNRS, LJAD, UMR 7351, Parc de Valrose, Nice 06108, France; ^3^CSEHS, Mohammed VI Polytechnic University, Ben Guerir, Morocco; ^4^INRIA Bordeaux Sud Ouest, Carmen Project, Talence, France; ^5^Department of Interventional Cardiology, Clinique des Spécialités Achifaa, Casablanca, Morocco

## Abstract

Fractional flow reserve (FFR) has proved its efficiency in improving patient diagnosis. In this paper, we consider a 2D reconstructed left coronary tree with two artificial lesions of different degrees. We use a generalized fluid model with a Carreau law and use a coupled multidomain method to implement Windkessel boundary conditions at the outlets. We introduce our methodology to quantify the virtual FFR and conduct several numerical experiments. We compare FFR results from the Navier–Stokes model versus generalized flow model and for Windkessel versus traction-free outlet boundary conditions or mixed outlet boundary conditions. We also investigate some sources of uncertainty that the FFR index might encounter during the invasive procedure, in particular, the arbitrary position of the distal sensor. The computational FFR results show that the degree of stenosis is not enough to classify a lesion, while there is a good agreement between the Navier–Stokes model and the non-Newtonian flow model adopted in classifying coronary lesions. Furthermore, we highlight that the lack of standardization while making FFR measurement might be misleading regarding the significance of stenosis.

## 1. Introduction

The coronary arteries are a common and important site of the development of sclerotic lesions. Thus, a detailed hemodynamic evaluation of the flow and its spatial and temporal distribution may give important insight to understand the pathology. In this view, the fractional flow reserve (FFR) plays a central role (see [1]). The fractional flow reserve (FFR) is an invasive measure that consists in introducing a pressure wire to a diseased artery to measure in vivo two values of blood pressure: the aortic pressure, *P*_aortic_, and the pressure distal to a lesion, *P*_distal_. These pressure values are then used to calculate the FFR ratio. According to the value obtained, the clinician decides whether the lesion is hemodynamically significant (FFR lower than 0.80) or nonsignificant (FFR higher than 0.80). In the case of a significant lesion, a revascularization is necessary. In this case, a realistic simulation of vascular blood flow inside the coronary arteries can be a better alternative to the invasive FFR (see [2–4]). On the one hand, a realistic blood flow simulation requires the use of an adequate flow model. For instance, Boujena et al. [5] presented a non-Newtonian flow model adapted to describe blood flow in the presence of atherosclerosis. Simulation in their paper was performed in 2D and 3D simplified geometries. On the other hand, the choice of suitable boundary conditions is crucial. In our previous paper [1], we presented a first virtual FFR estimation using the generalized fluid model in [5] and conducted different simulations to study the impact of the lesions parameters on the FFR value. However, we considered a simplified 2D geometry and reduced boundary conditions. In this paper, the domain of simulation corresponds to a realistic diseased coronary tree with many outlets. Thus, we address a special concern to the boundary condition model. In fact, the shape and the type of the function at the inlet are determinant of the flow and pressure patterns obtained in the domain. In the case where the study aims at comparing the results to in vivo measurements, the inlet boundary condition should be adequately chosen. Many works explored the effect of the inlet boundary condition; among them, Liu et al. [6] and Taylor and Steinman [7] presented realistic forms of inlet boundary condition in the case of coronary blood flow. Concerning the outlet, the most common boundary condition for blood flow corresponds to a constant pressure. However, this choice is not realistic when it comes to complex geometries, with many outlets. The strategy of resolution in this case consists in dividing the domain into two parts: the upstream domain and the downstream domain that includes the outlets. The outlet boundary conditions are defined in the downstream domain using an appropriate model, usually based on an electrical analogy, known as the Windkessel effect (see [8–10]). In the first section, we give the essential elements for simulation: the 2D multistenotic domain defined using segmentation techniques, the realistic flow model, and finally suitable boundary conditions. In the second section, we present the pressure and the flow distributions obtained for three different outlet boundary conditions. Finally, in the last section, we give an estimation of the fractional flow reserve (FFR) for two lesions using the pressure pattern in the stenotic coronary tree. The FFR calculation is performed using two different flow models: Navier–Stokes model and the generalized flow model, and considering diverse outlet boundary conditions.

## 2. Methods

### 2.1. Domain Definition: 2D Image Segmentation

**Figure 1:**
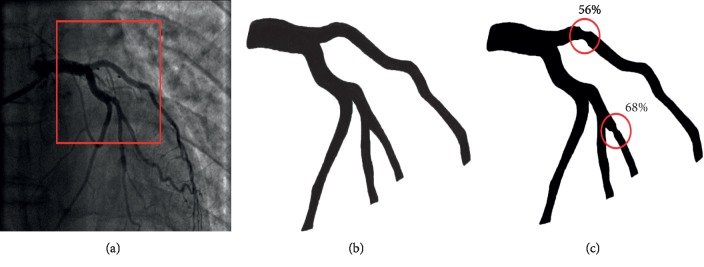
(a) The original angiography image (the coronary tree of interest is framed with red). (b) The black and white original image. (c) The resulting multistenotic coronary tree.

In order to create a realistic geometry for numerical simulation, we started from a 2D patient-specific angiography. An enhancement technique was done before this image could be segmented. In this phase, different filters were used to improve the contrast of the original image (see [11]). Then, opening/closing Matlab functions were used to extract a black and white image that contains only the coronary tree in which we are interested. It should be noticed that despite the fact that the original angiography corresponds to a stenotic coronary tree, due to the lower quality of the image and to the small degree of stenosis, the lesion could not appear in the black and white image. Since our aim in this paper is to investigate the impact of the flow model and the boundary conditions on the FFR, we introduced two different artificial lesions in the coronary tree. The first lesion corresponds to 68% stenosis and was drawn in the same location of the real patient's lesion. The second lesion corresponds to 56% stenosis and was drawn at the entrance of the longest branch in the coronary tree. This choice is justified by the purpose of calculating the fractional flow reserve in the case of traction free outlet boundary conditions. The resulting 2D multistenotic domain, the original extracted tree, and the original angiography are given in Figure 1.

Starting from the new multistenotic coronary tree, the segmentation and the meshing were performed later using a homemade FreeFem++ code (see [12]).

### 2.2. Coronary Blood Flow Model

The blood was assumed as an incompressible, non-Newtonian viscous fluid obeying the Carreau law with the viscosity shear rate relation given by

(1) $$\begin{array}{c} \mu =\mu_{\infty }+\left(\mu_{0}-\mu_{\infty }\right){\left(1+{\left(\lambda s\left(u\right)\right)}^{2}\right)}^{\left(n-1\right)/2}, \end{array}$$

where *μ*_0_=0.0456 Pa · s and *μ*_*∞*_=0.0032 Pa · s are the values of the viscosity for the lowest and highest shear rates. The parameter values *λ*=10.03 s and *n*=0.344 are typical for the Carreau law. The shear rate *s*(*u*) is defined as follows:

(2) $$\begin{array}{c} {\left(s\left(u\right)\right)}^{2}=2\;Du\;:Du=2\underset{i,j}{\sum }{\left(Du\right)}_{ij}{\left(Du\right)}_{ji}, \end{array}$$

with

(3) $$\begin{array}{c} Du=\frac{1}{2}\left(\nabla u+\nabla^{T}u\right). \end{array}$$

**Figure 2:**
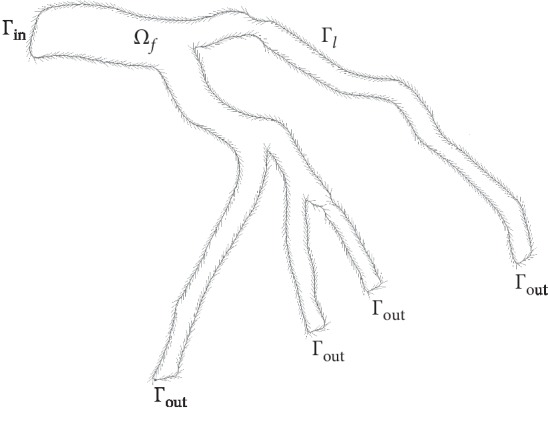
The 2D geometry considered. Arrows indicate the isoline orientation.

The geometrical 2D domain *Ω*_*f*_ is given in Figure 2. The time dependent two-dimensional generalized fluid equations presented in [5] were considered as the governing equations in the tree domain *Ω*_*f*_:

(4) $$\begin{array}{c} \left\{\begin{array}{cc} \rho_{f}\frac{\partial u}{\partial t}+\rho_{f}\left(u.\nabla \right)u-\nabla .\left(2\mu \left(s\left(u\right)\right)Du\right)+\nabla p=f, & \text{in }\Omega_{f}\times \left(0,T_{c}\right),\\ \nabla .u=0, & \text{in }\Omega_{f}\times \left(0,T_{c}\right),\\ 2\mu \left(s\left(u\right)\right)Du\;.n-pn=I, & \text{on }\Gamma_{in}\times \left(0,T_{c}\right),\\ u=0, & \text{on }\Gamma_{l}\times \left(0,T_{c}\right), \end{array}\right. \end{array}$$

where *u* is the incompressible velocity and *p* is the pressure.

*f* is the external body force applied to the fluid. *I* is the velocity function at the inlet that will be given in the next paragraph. In the computations, the blood density *ρ*_*f*_ was assumed to be constant at 1060 Kg · m^−3^. A no-slip condition was applied to the velocities at the lumen wall, considered to be inelastic and impermeable. A steady Stokes initial condition, with a Poiseuille function at the inlet was imposed. *T*_*c*_ corresponds to the duration of a cardiac cycle under normal conditions; we took *T*_*c*_=0.8 s (corresponding to a heart rate of 75 beats per minute).

### 2.3. Boundary Conditions: Inlet/Outlet

**Figure 3:**
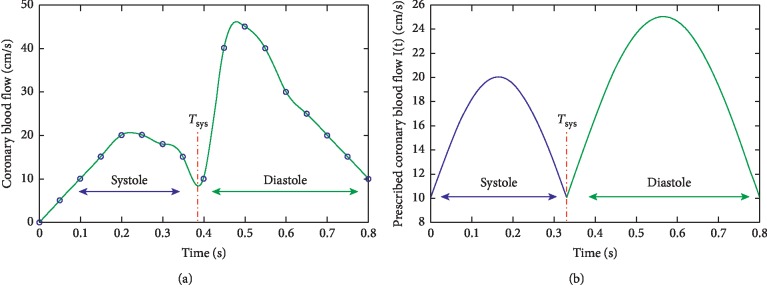
(a) Spline function approaching left coronary blood flow. (b) The flow function prescribed at the inlet *I*(*t*).

Since the processed image treated corresponds to a left coronary artery, we used sinusoidal functions to approach the inlet flow distribution into the left coronary artery. The shape of this function is well known (see [6]). Considering that *T*_sys_ is the period of systole, *t*_*s*_ the start of the systolic phase of the current cardiac cycle, and *t*_*d*_ the start of the diastolic phase, this periodic function *I*(*t*) can be written as follows:

(5) $$\begin{array}{c} I\left(t\right)=\left\{\begin{array}{cc} \left(I_{p}+I_{0}\;*\;\sin\left(\pi \;*\left(t-t_{s}\right)/T_{\text{sys}}\right),0\right), & 0\leq t\leq T_{\text{sys}},\\ \left(I_{p}+I_{c}\;*\;\sin\left(\pi \;*\left(t-t_{d}\right)/\left(T_{c}-T_{\text{sys}}\right)\right),0\right), & T_{\text{sys}}\leq t\leq T_{c}, \end{array}\right. \end{array}$$

where *I*_*p*_=10 cm/s represents the dominant flow, *I*_0_=10 cm/s, and *I*_*c*_=10 cm/s. *T*_sys_ is taken equal to 0.33 s. The remaining duration from the cardiac cycle corresponds to a diastole. The profile of this function is given in Figure 3.

To assess the influence of outlet boundary conditions on the pressure and flow fields, two different outlet boundary conditions were utilized in this study: traction free and a 2-element Windkessel model [9] to incorporate the resistant effect of the downstream bed. Indeed, the coupled multidomain method was utilized, as described in [9]. The idea is to couple the solution at the outflow boundaries of the computational domain of simulation with the 2-element Windkessel model (chosen in our case) to represent the downstream coronary vascular network cut from the real domain. It should be noticed that other so called lumped parameter models can be used for the downstream bed like 1D- or 2D-based impedance boundary conditions (see [9] or [10] for further details).

To couple the values of *u* and *p* between the upstream domain (extracted tree of interest) and the downstream domain, we need to introduce the two operators: $M=\left[M_{m},\overset{⟶}{M_{c}}\right]$ and $H=\left[H_{m},\overset{⟶}{H_{c}}\right]$. *M* represents the traction while *H* represents the flow at each coronary outlet. Each one of *M* and *H* is composed of a momentum and a continuity operator, respectively.

For each coronary outlet, we define the operators *M* and *H* by replacing the coronary outlet pressure *P*(*t*) with the ordinary differential equation obtained from the 2-element Windkessel model. In our case, the same model is used to represent all the outlets. The variational formulation of our problem in this case can be written as follows:

(6) $$\begin{array}{c} \left\{\begin{array}{cc} \rho_{f}\int_{\Omega_{f}}\frac{\partial u}{\partial t}v\text{d}x+\left(Au,v\right)+\rho_{f}b\left(u,u,v\right)-\int_{\Gamma_{\text{out}}}v\cdot \left(M_{m}\left(u,p\right)+H_{m}\left(u,p\right)\right)\cdot \overset{⟶}{n}\text{d}s+\int_{\Gamma_{\text{out}}}q\cdot \left(\overset{⟶}{M_{c}}\left(u,p\right)+\overset{⟶}{H_{c}}\left(u,p\right)\right)\cdot \overset{⟶}{n}\text{d}s=\int_{\Gamma_{\text{in}}}I\text{d}s, & \forall v\in V,\forall p\in P,\\ u\left(0\right)=u_{0}, & \text{in }\Omega_{f}, \end{array}\right. \end{array}$$

with

(7) $$\begin{array}{c} \left(Au,v\right)=\int_{\Omega_{f}}2\mu \left(s\left(u\right)\right)Du\;:Dv\text{ d}x,\\ b\left(u,v,w\right)=\overset{2}{\underset{i,j=1}{\sum }}\int_{\Omega_{f}}u_{i}\frac{\partial v_{j}}{\partial x_{i}}w_{j}\text{d}x. \end{array}$$

The expressions of the boxed terms representing the downstream bed physics will be given in the next section.

#### 2.3.1. Windkessel Model

**Figure 4:**
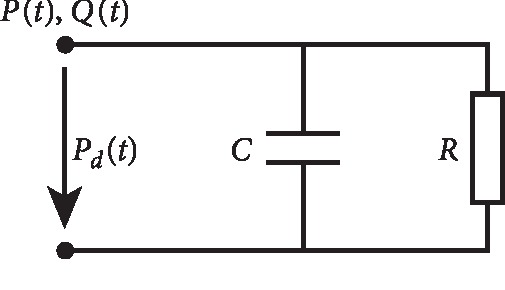
Windkessel electrical analogy.

Lumped parameter models were originally derived by the physiologist Otto Frank in an article published in 1899 [13] to describe the afterload of the heart related to pumping blood through the arterial system, as described in [9]. The Windkessel model is based on an electrical analogy where an arterial tree is assimilated to an electric circuit. The parameters of the components of the circuit (resistances, capacitances, etc) correspond to the properties of each branch. The variables are the voltage at every node and the current in each branch. In the context of blood flowing in an arterial network, pressure plays the role of voltage and flow rate the role of current. During a cardiac cycle, a 2-element Windkessel model takes into account the effect of arterial compliance and total peripheral resistance. In the electrical analogy, the arterial compliance (*C* in cm^3^/mmHg) is represented as a capacitor with electric charge storage properties. Peripheral resistance of the systemic arterial system (*R* in mmHg s/cm^3^) is represented as an energy dissipating resistor. The flow of blood in the heart (*Q*(*t*) in cm^3^/s) is analogous to that of current flowing in the circuit and the outlet blood pressure (*P*(*t*) in mmHg) is modeled as a time-varying electric potential. We also consider the downstream intramyocardial pressure *P*_*d*_: the pressure in the left atrium.  Figure 4 gives a schematic view of the representative circuit of the dynamics in each compartment of the coronary tree. The resulting differential equation can be written as follows:

(8) $$\begin{array}{c} Q\left(t\right)=\frac{P\left(t\right)-P_{d}\left(t\right)}{R}+C\frac{\text{d}\left(P-P_{d}\right)\left(t\right)}{\text{d}t}. \end{array}$$

Using the operators *M* and *H*, we couple the flow and pressure at each coronary outlet between the upstream finite element model and the downstream Windkessel model. As proved in [9], their expressions are obtained by solving the ordinary differential equation given in equation (8).

(9) $$\begin{array}{c} \int_{\Gamma_{\text{out}}}v\cdot M_{m}\left(u,p\right)\cdot \overset{⟶}{n}\text{d}s=-\int_{\Gamma_{\text{out}}}v\cdot \overset{⟶}{n}\left(R\int_{\Gamma_{\text{out}}}u\cdot \overset{⟶}{n}\text{d}s+\int_{0}^{t}\frac{e^{-\left(t-t_{1}\right)}/\delta }{C}\int_{\Gamma_{\text{out}}}u\left(t_{1}\right)\cdot n\text{d}s\cdot \text{d}t_{1}+\overset{⟶}{n}.\tau .\overset{⟶}{n}\right)\text{d}s+\int_{\Gamma_{\text{out}}}v\cdot \tau \cdot \overset{⟶}{n}\text{d}s,\\ \int_{\Gamma_{\text{out}}}v\cdot H_{m}\left(u,p\right)\cdot \overset{⟶}{n}\text{d}s=-\int_{\Gamma_{\text{out}}}v\cdot \overset{⟶}{n}\left(\left(P\left(0\right)-R\int_{\Gamma_{\text{out}}}u\left(0\right).\overset{⟶}{n}\text{d}\Gamma -P_{d}\left(0\right)\right)e^{-t/\delta }+P_{d}\left(t\right)\right)\text{d}s,\\ \overset{⟶}{M_{c}}\left(u,p\right)=u,\\ \overset{⟶}{H_{c}}\left(u,p\right)=\overset{⟶}{0}, \end{array}$$

where *δ*=*RC*, *R*=0.95, and *C*=1.06.

We consider the same values of Windkessel parameters for all outlets. The downstream pressure *P*_*d*_ is also varying in time. The expression of *P*_*d*_ can be found by solving analytically differential equation (8) considering a simplified expression for *Q*(*t*), based on common learning of the cardiac physiology. During diastole, when the ventricules are relaxed, there is no blood flow in the aorta. However, with ventricular contraction during systole, blood is injected into the aorta and can be modeled as a sinusoidal wave. In this work, we use the same approach as in [8] to implement *P*_*d*_(*t*) in the case of a 2-element Windkessel model. The velocity and pressure fields inside the realistic computational domain were solved semi-implicitly, due to the viscosity term with Carreau law involving shear rate *s*(*u*). The finite element method for the resolution was implemented under FreeFem++. The numerical results obtained are presented in the next sections.

### 2.4. Fractional Flow Reserve (FFR)

The fractional flow reserve is crucial to quantify the hemodynamic severity of the stenosis in the case of intermediate lesions, where the degree of stenosis varies between 40% and 70% (see [14]). From a clinical standpoint, this measure indicates the degree of implication of stenosis in ischemia (a deficient supply of oxygen to the myocardium).

**Figure 5:**
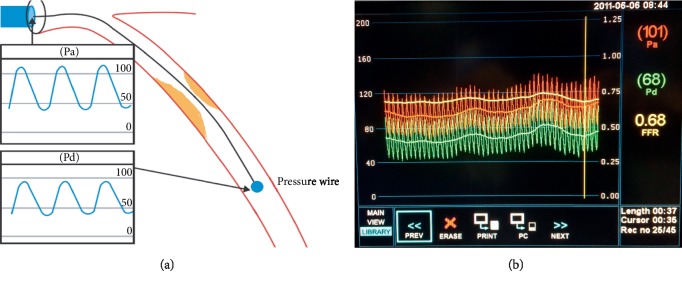
(a) Schema of the invasive FFR technique. (b) A typical example of FFR measurement. Automated calculation of FFR (yellow) corresponds to the ratio of mean distal coronary pressure (green) to mean aortic pressure (red) during maximal hyperemia.

Like presented in our previous paper [1], to measure the fractional flow reserve (FFR) during the invasive test, the operator crosses the coronary lesion with an FFR-specific guide wire. This guide wire is designed to record the coronary arterial pressure beyond the lesion (Figure 5(a)). Once the transducer is distal to the lesion (approximately 20 mm), a hyperemic stimulus is administered by injection through the guiding catheter. The maximal hyperemia should be reached to avoid underestimating the value of FFR (see [15]). The mean arterial pressures from the pressure wire transducer *P*_aortic_ and from the guide sensor *P*_distal_ are then used to calculate FFR ratio: FFR=*P*_distal_/*P*_aortic_ (Figure 5(b)). The aortic pressure *P*_aortic_ is the central blood pressure at the root of the aorta, while the distal pressure *P*_distal_ corresponds to the pressure at the surface of the sensor (pressure wire in Figure 5(a)). Both pressures given by the FFR instrument are calculated as a temporal mean, over the cardiac cycle, of pressures *p*_*s*_(*t*) captured at each frequency drop (see [1]). These pressures can be written as follows:

(10) $$\begin{array}{c} P=\frac{1}{T_{c}}\int_{0}^{T_{c}}p_{s}\left(t\right)\text{d}t. \end{array}$$

An FFR value lower than 0.75 indicates a hemodynamically significant lesion. An FFR value higher than 0.8 indicates a lesion that is not hemodynamically significant. Values between 0.75 and 0.80 are critical. In this case, the FFR is not a reliable element in clinical decision making.

Our objective is to give an estimation of the FFR for both lesions in the diseased coronary tree using the pressure distributions obtained. We aim at studying the effect of the flow model and the outlet boundary conditions on the FFR value. We consider two different flow models: Navier–Stokes model versus the generalized flow model (presented in the previous section), and three options for outlets boundary conditions: Windkessel, traction-free outlets, and mixed boundary conditions given in detail in the next section. We assume that the 2D geometry for FFR measurements corresponds to a maximal vasodilation. In fact, a clinically usable FFR value (to be compared to real FFR measurements) is not our ultimate goal in this paper. We implement an algorithm to compute a virtual FFR following the same calculation strategy as used by the clinical FFR device, like in [1]. At each time step, the aortic pressure *P*_*a*_ is calculated by the mean pressure of the points at 1 cm from the inlet of the coronary tree, in order to avoid all the transient effects at the entrance.

**Figure 6:**
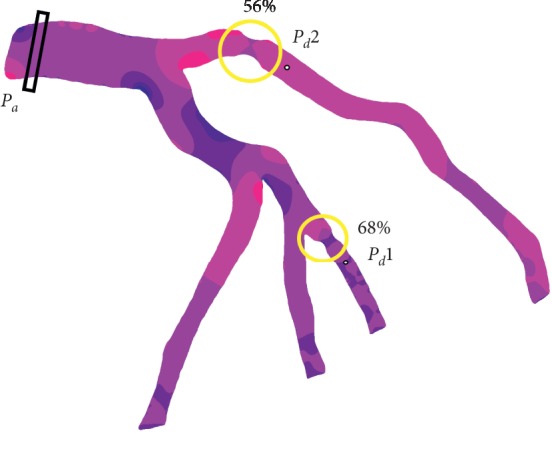
FFR calculation. The mutlistenotic coronary tree contains two lesions: 56% stenosis and 68% stenosis.

The distal pressure *P*_*d*_ is obtained at a distance of 1 cm beyond each lesion on the sensor contour assimilated to a disk with constant diameter. The ratio between the sensor diameter and the reference diameter of the branch is *D*_sensor_/*D*_ref_=1/10, based on the common magnitude of the sensor diameter that is 0.014^″^=0.35 mm. It should be noticed that the 2D disk is not virtual and is considered as an obstacle to the flow, in contrast to the virtual box for *P*_*a*_ calculation. The diagram in Figure 6 describes the approach. At each cardiac cycle—and during five consecutive cardiac cycles—a temporal mean pressure of *P*_*a*_ and *P*_*d*_ is performed. The ratio of these two pressures gives an FFR value at each cardiac cycle.

## 3. Numerical Results

Simulations are performed using the finite element solver FreeFem++, based on a semi-implicit time discretization scheme. Fluid velocity and pressure are calculated at each time step. The time step used is *δt*=5.10^−3^ s and the duration of a cardiac cycle is *T*_*c*_=0.8 s. Five consecutive cardiac cycles were simulated to reach a periodic regime of the flow. As for the spatial discretization, we use a 55353 elements mesh. To study the dependency of the solution on the numerical mesh, we perform a mesh refinement convergence study for FFR estimation. The results from this convergence study are presented in Table 1 in Section 4.1.

### 3.1. Comparison of Outlet Boundary Conditions

**Figure 7:**
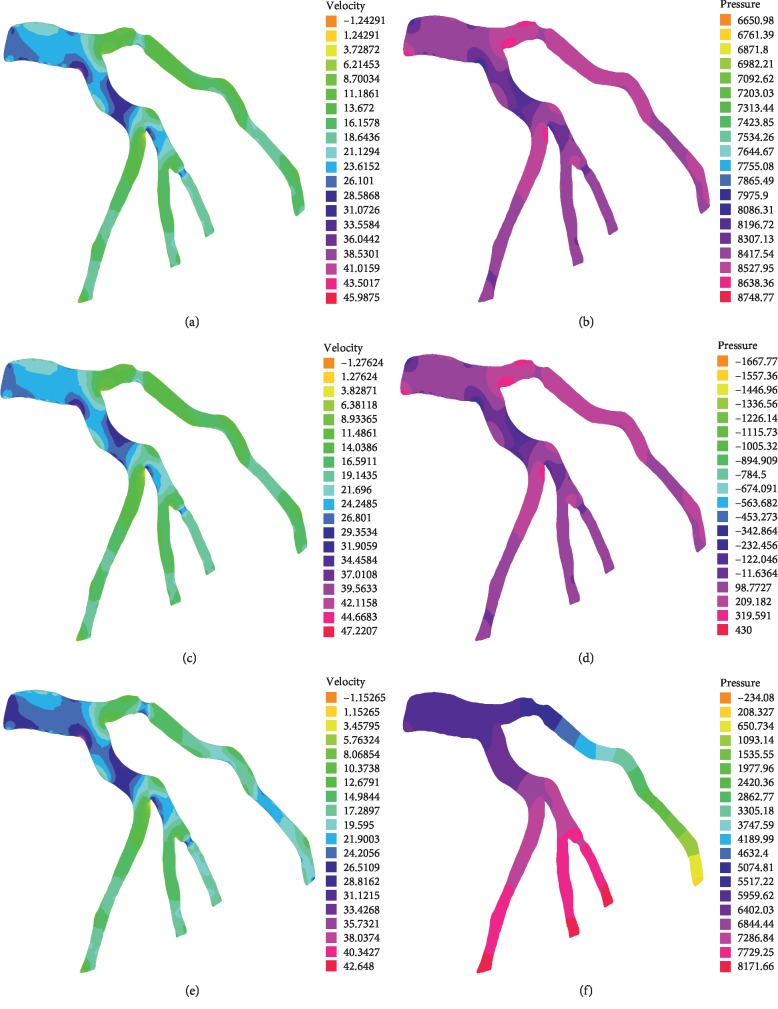
Velocity fields at peak diastole using generalized fluid model with Windkessel outlet BCs (a), free outlet BCs (c), and mixed outlet BCs (e). Corresponding pressure fields: Windkessel outlet BCs (b), free outlet BCs (d), and mixed outlet BCs (f).

The results in Figure 7 give the flow (magnitude of velocity) and pressure patterns into the stenotic coronary tree at the peak diastole of the fifth cardiac cycle. The same flow model—non-Newtonian Navier–Stokes model—is used for all simulations. However, three different outlet boundary conditions were considered: firstly, we considered that all outlets correspond to a 2-element Windkessel model. Secondly, we used a free traction boundary condition for all the outlets. Finally, we introduced mixed outlet boundary conditions where the longest stenotic branch of the tree is considered as a traction-free outlet, and the remaining three branches correspond to 2-element Windkessel models with the same parameters.

We can see that the velocity and pressure fields have approximately the same layout with Windkessel and traction-free outlet boundary conditions even if the isovalues are different. This is due to the fact that in both cases, no one of the outlets is advantageous compared to the others: resistive effect or traction free in all exits, especially that the Windkessel model adopted uses the same parameter values for all outlets. In contrary, with mixed boundary conditions, the longest branch is free while the remaining corresponds to a 2-element Windkessel model. As a result, we observe lower values of pressure in this branch and eventually higher values of velocity which is completely intuitive.

### 3.2. Flow Distributions: Navier–Stokes Model vs. Non-Newtonian Model

**Figure 8:**
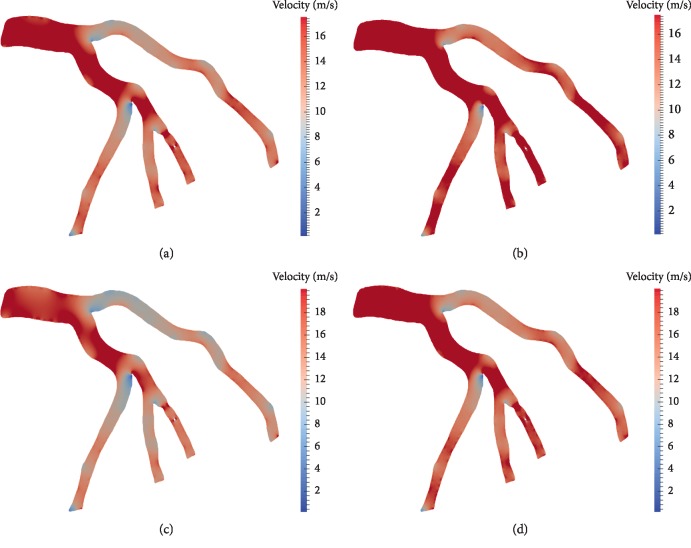
Velocity field using generalized fluid model at peak systole (a) and peak diastole (b). Velocity field using Navier–Stokes model at peak systole (c) and peak diastole (d).

The plots in Figure 8 give velocity fields at different times of the cardiac cycle, in particular, at peak systole and peak diastole. The simulations were performed with both flow models: Navier–Stokes model (Figures 8(b) and 8(d)) and non-Newtonian fluid model (Figures 8(a) and 8(c)). The same type of boundary conditions is considered for all simulations: a 2-element Windkessel model for all outlets.

Values of blood velocity vary from 2 m/s to 19 m/s, and we can clearly observe that the values given by the Navier–Stokes model are higher than those given by the non-Newtonian flow model. This is due to the viscosity term that is constant in the Navier–Stokes model in contrary to the non-Newtonian fluid model where the viscosity varies according to the Carreau law introduced in Section 2.2.

The results are well illustrating the difference between Newtonian and non-Newtonian rheologies. In our case, the non-Newtonian flow model is more adapted to simulate the flow. Firstly because the vessels' caliber in coronary arteries is small comparing to the aorta for example, for which Navier Stokes model is widely used. In this case, we cannot neglect the non-Newtonian behaviour of blood that is composed not only of plasma (that can be assimilated to a Newtonian fluid) but also of blood cells that are the main factors behind blood viscosity. More precisely, the frictions between them and against the arterial wall are more important when the vessel's diameter is small.

**Figure 9:**
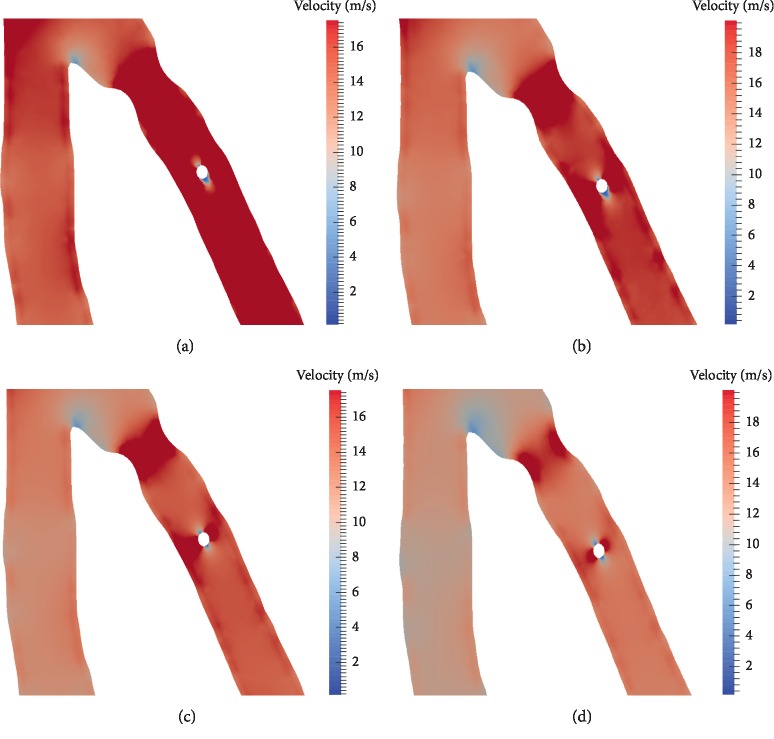
Velocity distributions near stenosis with the two flow models at different times of the cardiac cycle. (a) Peak diastole—generalized flow model. (c) Peak systole—generalized flow model. (b) Peak diastole—Navier–Stokes model. (d) Peak systole—Navier–Stokes model.

We also observe from Figure 8 that the values of velocity are higher at peak diastole than at peak systole for both models. The flow simply follows the profile given at the inlet of the coronary tree, given in Section 2.3.  Figure 9 corresponds to velocity fields near stenosis and around the obstacle: the sensor wire in this case assimilated to a disk in 2D. Shear stresses are observed to be higher with the non-Newtonian flow model than with the Navier–Stokes model. FFR values obtained with both models are 0.76 with the Navier–Stokes model vs. 0.747 with the non-Newtonian flow model. The difference between the value given by the two models is still small which means that the lesions are classified in the same value range: hemodynamically important.

## 4. Fractional Flow Reserve (FFR) Computation

The lesions of interest have a degree of stenosis equal to 56% and 68%, which makes them both in the intermediate value range. This justifies the necessity of a recourse to an estimation of the fractional flow reserve in taking a clinical decision.  Table 2 gives the FFR values for these two lesions using the Navier–Stokes model and the generalized flow model and considering three different options for the outlet boundary conditions: Windkessel model, traction-free outlets, and mixed outlet boundary conditions where only the longest branch is considered free while the other branches' outlets are assimilated to a 2-element Windkessel model. The FFR result in Table 2 corresponds to the average FFR value over five cardiac cycles in each different case of study.

The model used for simulation is nonlinear, as shown in equation (4). Moreover, the domain of simulation is not a plane geometry but a curved boundary configuration, and the finite element method used is standard (triangular elements are not well adapted to curved domain in the opposite of isoparametric elements, for example, see [16, 20]). As a result, the solution of our system is dependent on the numerical mesh and the FFR estimation algorithm is sensitive to the mesh discretization (as shown in Table 2). In this case, the accuracy of the FFR estimation might be questioned. In order to study the sensitivity of the FFR value computed, a mesh refinement convergence study is presented in the next section. Then, a discussion of the results in Table 2 is provided in Section 4.2, based on the finer mesh simulations.

### 4.1. Convergence Study

In this section, we lead a mesh refinement study for FFR computation. We consider 10 different meshes, with a number of elements varying from 1478 to 51528. We use P2 element for velocity components and P1 element for the pressure. For each finite element space considered, the simulation was run during five consecutive cardiac cycles; we compute the FFR value and the average FFR value FFR_*a*_ over the previous cycles. We only considered one lesion, that is, lesion 1 represented in Figure 6. The time step d*t*=5 × 10^−3^ considered for all simulations was small enough so that the numerical stability of the semi-implicit scheme is verified. For all simulations, we use the non-Newtonian model for flow and the 2-element Windkessel boundary condition for all outlets. The main results from this study are presented in Table 1.

We can observe from the results in Table 1 that from the third cycle the FFR average FFR_*a*_ is not subject to a big change (two decimal places constant) for all the space discretizations considered. We can see that for the two final meshes, the FFR_*a*_ value is not subject to a big change. The value of 0.90 can be adopted to make a clinical decision. This lack in the accuracy of the estimation can only affect the lesions for which the FFR value obtained is close to the clinical FFR cutoff. In general, for these special cases, the practitioner resorts to the patient clinical history and to some additional tests to decide for the strategy of treatment (see [14]).

### 4.2. Discussion

The flow model considered for simulations is only slightly influencing the FFR value. For example, considering the possible options for outlets boundary conditions, the difference in the FFR between the Navier–Stokes model and the non-Newtonian flow model does not exceed 2% where the outlets are not all traction free. In the case of free outlets, the decrease in the FFR value for the first lesion is quite surprising (cells in grey in the table): up to 79% and 75% with the generalized fluid and the Navier–Stokes models, respectively. In fact, there is a huge pressure drop in the *P*(*t*) value since the distal sensor for this lesion is not far enough from the free exit. On the contrary, we do not have this problem with the second lesion as the branch is long enough beyond the sensor. This shows that these types of boundary conditions are not appropriate and not realistic to perform such calculation in the coronary arteries, though their widespread use (see [17]). Now, comparing Windkessel and mixed boundary conditions, we can see that the first lesion conserves the same FFR classification—hemodynamically non significant—while the second lesion moves from the non significant stenosis class to the significant one. These same classifications are conserved with both flow models. Considering the fact that the first lesion has an important degree of stenosis (68%) while the second one is a 56% lesion, this result confirms that the FFR value is not only depending on the degree of stenosis which renders a physical severity of the lesion but also on the hemodynamical flow inside the connected tree, strongly impacted by the flow model and the nature of boundary conditions (inlet and especially outlet boundary conditions).

## 5. Quantification of the Sensor Position Impact on the FFR Value

### 5.1. Sensor Position: Effect on the Virtual FFR

**Figure 10:**
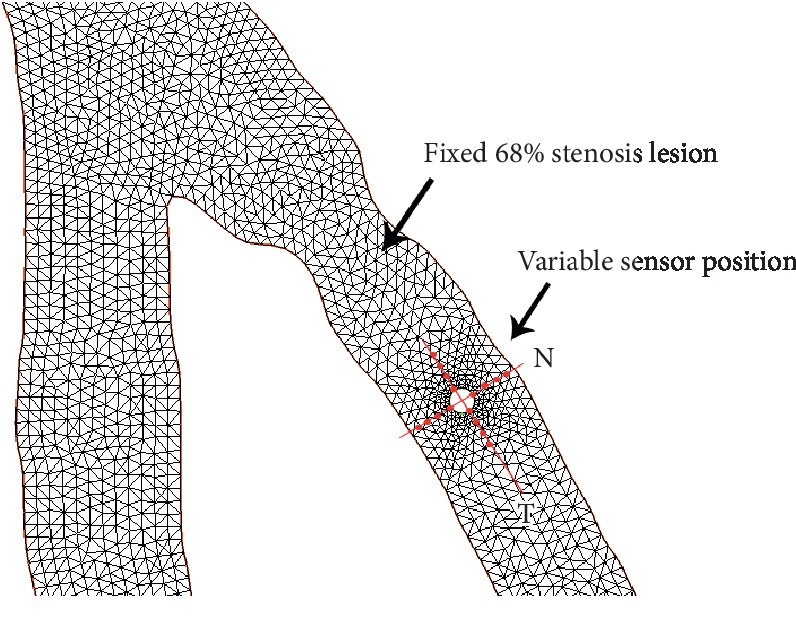
Distal sensor displacement according to the normal and tangential positions.

FFR measure, as explained previously, uses pressure sensor-tipped intracoronary wires to quantify the transtenotic pressure gradient *P*_*d*_. In this section, we aim to study the error induced by the deviation of the sensor from its position of origin that is in general defined at the center of the branch cross section (see Figure 10).

The mean value of the distal pressure *P*_*d*_ is measured using the sensor's contour points at each time step. In this 2D study, we consider only two sources of error, that is, sensor displacement along the flow direction (vector *T*) and along the normal to flow direction (vector *N*) (see Figure 10).

The common value for FFR sensors diameter is 0.014 inches, which corresponds to 0.35 mm. The same size of FFR sensors is used in almost all clinical centers for standardization reasons, which is why the sensor's probe size was not considered as a source of error.

We incorporate a 2D disk in a position of reference inside the coronary tree, given in Figure 10. We consider that this position is subject to variation due to the randomized aspect of the clinical intervention: the practitioner is not very precise as to the sensor position and two different practitioners can adopt two different sensor's positions for the test, which may modify the FFR value. The sensor's position can also vary due to the flow during the measure. Figure 10 shows how the sensor position can vary in the normal and tangential directions. The disk diameter is considered constant, and the ratio between the sensor diameter and the reference diameter of the branch is *D*_sensor_/*D*_ref_=1/10.

The aortic pressure *P*_*a*_ is calculated virtually at 1 cm from the entrance of the main branch of the arterial tree. In order to quantify the effect of the flow model and the outlet boundary conditions on the FFR value in presence of the distal sensor, we conducted different simulations. On the one hand, we compared the Navier–Stokes model and the generalized flow model. On the other hand, a comparison between traction-free outlet boundary conditions and the Windkessel model is presented. From the two artificial lesions introduced in the previous sections, only one was considered for each study.

### 5.2. FFR Variation Corresponding to Both Directions

**Figure 11:**
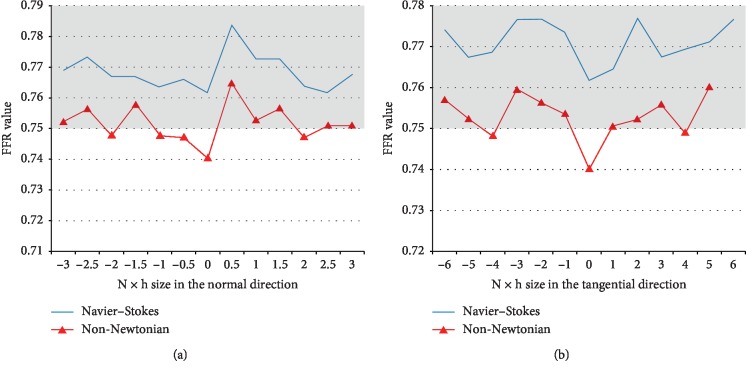
(a) Comparison between FFR values for Navier–Stokes model and non-Newtonian flow model obtained by moving the sensor in the normal direction. (b) Comparison between FFR values for Navier–Stokes model and non-Newtonian flow model obtained by moving the sensor in the tangential direction. The grey area represents critical FFR values.

The following graphics in Figure 11 illustrate the variations in the FFR value for the fixed 68% stenotic lesion. In each simulation, a different position of the sensor is considered.  Figure 11(a) represents a variation of the FFR value according to the normal to flow direction *N* (see Figure 10), and the tangential coordinate is fixed while the normal varies from −3/10 × *D*_ref_ to 3/10 × *D*_ref_. *D*_ref_ is the diameter of reference of the stenotic branch.  Figure 11(b) represents the variation of the FFR value according to the flow direction *T* (Figure 10). The normal coordinate is fixed to the center of the branch while the tangential varies from −6/10 × *D*_ref_ to 6/10 × *D*_ref_. For each position of the sensor, two simulations are run: one with the Navier–Stokes model and the other with the non-Newtonian flow model, to obtain two values of the virtual FFR, represented, respectively, in the blue and red curves (Figure 11). The same space discretization is used for all simulations, based on a 55353 elements mesh file. In the two figures, a grey area is drawn to represent the critical zone for FFR values, and the cutoff considered in this case is 0.75. We can see that with the two models, for each new positions of the sensor, the virtual FFR illustrates important variations. With the Navier–Stokes model (blue curve), the lesion was classified in the same value range for all the positions of the distal sensor: not hemodynamically significant. On the contrary, with the generalized flow model, 8/26 positions of the distal sensor classified the lesion to be hemodynamically significant while the remaining positions gave the opposite conclusion. In all simulations, the same model for boundary conditions was considered: a 2-element Windkessel model as introduced in Section 2.

**Figure 12:**
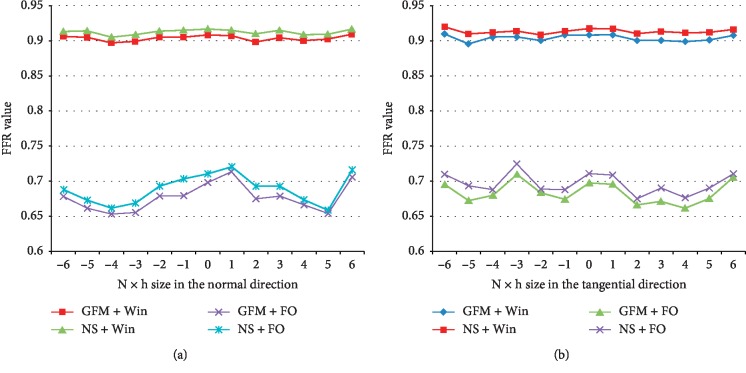
(a) Comparison between FFR values for Navier–Stokes model and non-Newtonian flow model combined with free outlets or Windkessel BC obtained by moving the sensor in the normal direction. (b) Comparison between FFR values for Navier–Stokes model and non-Newtonian flow model combined with free outlets or Windkessel BC obtained by moving the sensor in the tangential direction.

The graphics from Figure 12 illustrate the effect of boundary conditions on the virtual FFR computed. These simulations combine in each time a different flow model: Navier Stokes vs. non-Newtonian flow model and different boundary conditions: Windkessel model vs. free outlets BC, though this last choice is not very realistic, as proved by the results in Table 2. The FFR values represented in Figure 12 correspond to the 56% stenotic lesion, given in Figure 6.

The main reason to adopt a different lesion in this case is the fact that this last is positioned at the entrance of the branch and that this branch is long enough to keep the distal sensor far from the exit. This condition is not verified in the first lesion. Otherwise, we obtain an important pressure drop when free outlet boundary conditions are considered. We can see from the curves that for both flow models and for sensor's displacement in both directions, the lesion is classified nonsignificant if the outlet boundary conditions are Windkessel: virtual FFR beyond 0.85. However, this lesion becomes significant when considering free outlet boundary conditions: virtual FFR under 0.75.

This first 2D approach is adopted to illustrate the variability of the computed FFR due to the eventual perturbations in the distal sensor's position which make it possible to obtain two contradictory clinical conclusions for the same lesion if the circumstances of the FFR intervention are different. This arbitrary position of the distal sensor can be in the origin of drift that is unavoidable during FFR measurement.

## 6. Conclusions and Perspectives

In this work, we calculated the fractional flow reserve (FFR) corresponding to a multistenotic patient-specific coronary tree. The two lesions of interest were not present in the original angiography but were artificially incorporated inside the vascular tree. Thus, the used geometry is sufficiently realistic to represent important features of the flow in a real diseased coronary tree. The two intermediate lesions of interest have degrees of stenosis of 68% and 56%. The strategy of FFR computation was based on a stabilized semi-implicit time discretization scheme of the nonlinear problem, using triangular elements. A sensitivity study was performed to study FFR sensitivity to mesh discretization. The FFR classification for these two lesions was not influenced by the flow model adopted for the simulation even if the FFR values were slightly different between the Navier–Stokes model and the non-Newtonian flow model. However, according to the chosen option for outlet boundary conditions, we could obtain different lesions classifications. Based on the finer mesh simulations, the second lesion moved from the insignificant to the significant value range stenosis. We can summarize the following conclusions:

- There is a good agreement between the Navier–Stokes model and the non-Newtonian flow model in simulating coronary blood flow and thus in classifying coronary lesions if the fluid parameters are carefully chosen (see [18]).
- Traction-free outlet boundary conditions are not realistic to consider for FFR computation, since they are sensitive to the FFR sensor position. Moreover, they do not reproduce the dynamics of the coronary downstream bed, which is in general removed from the geometry (opposite to Windkessel model or other lumped parameter models, see [9, 10]).
- The study confirms the fact that the degree of stenosis is not enough to quantify the severity of a lesion (see [19]). In our case, the two considered lesions had different classifications each time outlet boundary conditions were modified.

Our aim was to place emphasis on the sensitivity of virtual FFR calculations and flow features in coronary arteries to the physical model, the boundary conditions, and the space discretization as well, keeping out of scope the important purpose of validating virtual FFR against clinical data. Indeed, the FFR value issued from a 2D simulation cannot be directly compared to the real invasive FFR, since a 2D angiography-based reconstruction of the coronary tree is not the best representation of the physiological domain. As a result, one perspective to this work is the reproduction of the coronary blood flow into a 3D geometry (see [20]). During FFR invasive measurement, many undesirable effects can occur during the procedure, leading to a drift in the value of the index. In this chapter, we aimed to illustrate one of these effects through the uncertainty in the pressure distal sensor's position. Using a 2D modelling of the FFR measurement scenario with a similar computation strategy to the clinical device, we could demonstrate only by simulation that for the same lesion we can have different medical conclusions. We conducted different simulations using two fluid models for blood, Navier–Stokes model and non-Newtonian model, and different outlet boundary conditions: Windkessel vs. free outlets. All the considered cases in 2D showed that FFR value is subject to small changes that amplify depending on the degree of stenosis, which affects the accuracy of the measure. The study presented in this work is an evident proof that simulation is an essential key to explore all the eventual sources of error that might occur during FFR test and that might impact the accuracy of the measure. The developed methods can provide clinicians with powerful new tools, rivaling and even surpassing experimental methods to investigate the mechanisms of disease and to design new medical devices and therapeutic interventions.

## Figures and Tables

**Table 1:** FFR values for the second lesion at 5 different cardiac cycles, for different values of the mesh size.

*N* vertices	Cycle *N*	1	2	3	4	5
1478	FFR	0.9803	0.93115	0.8538	0.9179	0.9203
	FFR_*a*_	0.9803	0.9557	0.9217	**0.9208**	**0.9207**

1746	FFR	0.9792	0.9382	0.9128	0.9278	0.9505
	FFR_*a*_	0.9792	0.9587	0.9434	**0.9420**	**0.9417**

2143	FFR	0.9824	0.9228	0.8644	0.9308	0.9111
	FFR_*a*_	0.9824	0.9526	0.9232	**0.9251**	**0.9223**

2786	FFR	0.9795	0.9214	0.8777	0.9138	0.9221
	FFR_*a*_	0.9795	0.9504	0.9262	**0.9231**	**0.9229**

3731	FFR	0.9819	0.9203	0.8180	0.9210	0.9143
	FFR_*a*_	0.9819	0.9511	0.9067	**0.9103**	**0.9111**

5254	FFR	0.9759	0.9194	0.8037	0.9014	0.9101
	FFR_*a*_	0.9759	0.9476	0.8996	**0.9001**	**0.9021**

7908	FFR	0.9790	0.9267	0.8305	0.9082	0.9106
	FFR_*a*_	0.9790	0.9528	0.9120	**0.9111**	**0.9110**

13916	FFR	0.9724	0.9231	0.8251	0.9222	0.9172
	FFR_*a*_	0.9724	0.9477	0.9068	**0.9107**	**0.9120**

30658	FFR	0.9619	0.9243	0.8278	0.9064	0.9036
	FFR_*a*_	0.9619	0.9431	0.9046	**0.9051**	**0.9048**
51528	FFR	0.9608	0.9220	0.8271	0.8993	0.9013
	FFR_*a*_	0.9608	0.9414	0.9033	**0.9023**	**0.9021**

The same value of time step was adopted for all simulations d*t*=5 × 10^−3^. FFR is the value for the cardiac cycle while FFR_*a*_ is the average FFR value.

**Table 2:** FFR values for both lesions corresponding to the two flow models and the different outlet boundary conditions. Two different mesh files were used for these calculations: coarse mesh (14240 elements) and fine mesh (55353 elements).

Mesh size	Flow model	Outflow BC	FFR 1	FFR 2
Coarse mesh	Navier–Stokes	Windkessel	0.917	0.760
		Free outlets	0.710	0.119
		Mixed BC	0.717	0.885
	Generalized flow	Windkessel	0.908	0.7478
		Free outlets	0.698	0.106
		Mixed BC	0.722	0.8567

Fine mesh	Navier–Stokes	Windkessel	0.9515	0.8205
		Free outlets	0.8704	0.2459
		Mixed BC	0.7172	0.9891
	Generalized flow	Windkessel	0.9404	0.8039
		Free outlets	0.8096	0.2082
		Mixed BC	0.7229	0.9791

## Data Availability

No data were used to support this study.
